# Prevalence of symptoms of sleep apnoea syndrome in Burkina Faso

**DOI:** 10.7196/AJTCCM.2019.v25i2.006

**Published:** 2019-07-31

**Authors:** G Ouédraogo, A R Ouédraogo, A S Adambounou, K Boncoungou, S Maiga, R Koalga, R Nacanabo, A Tiendrebeogo, G Badoum, M Ouédraogo

**Affiliations:** 1 Department of Pulmonology, University Hospital Yalgado Ouédraogo, Ouagadougou, Burkina Faso; 2 Department of Pulmonology, University Hospital Sylvanus Olympio, Lomé, Togo; 3 Department of Pulmonology, Regional Hospital of Ouahigouya, Burkina Faso

**Keywords:** prevalence, apnea, sleep, snoring, daytime sleepiness

## Abstract

**Introduction:**

Sleep apnoea syndrome (SAS) is a frequent and underdiagnosed pathology. Epidemiological studies in sub-Saharan Africa are few. Our study aimed to determine the prevalence of SAS symptoms in an adult population in Burkina Faso.

**Methods:**

A cross-sectional study whose data collection took place at the Yalgado Ouédraogo Teaching Hospital, from 1 September to 31 October 2014. We randomly enrolled all subjects aged at least 25 accompanying an outpatient t the time of a visit. A strong suspicion of SAS was established for every combination of ordinary snoring with excessive daytime sleepiness and/or sleep apnoea.

**Results:**

The study included 311 subjects – 181 men and 130 women. The mean (standard deviation (SD)) age was 31.84 (8.25) years and the average (SD) BMI was 23.14 (3.67) kg/m². The prevalence of excessive daytime sleepiness, snoring and sleep apnoea was 4.5%, 26% and 9.6%, respectively. A strong suspicion of SAS was found in 9.6% of respondents and the risk factors associated with this strong suspicion were BMI≥25 kg/m² (odds ratio (OR) 2.7; p=0.012), and poor-quality sleep (OR 3.7; p<0.001).

**Conclusion:**

A significant proportion of our sample had symptoms suggestive of SAS. Testing with either respiratory polygraphy or polysomnography should be proposed to the presumptive cases for early diagnosis and treatment.

## Background

Sleep apnoea syndrome (SAS) is a common pathology of the middle-aged adult.^[1]^ It is under diagnosed because it is poorly understood by both the general population and healthcare workers. It is a serious condition, mainly because of its neurocardiovascular (hypertension, cardiovascular disease, stroke), metabolic (diabetes, dyslipidemia, metabolic syndrome) and socio-occupational complications (road accidents, decreased quality of life).^[2–8]^ In Western countries, it is classified as a public health problem, with the prevalence varying from 3% to 28% in the adult population.^[1]^ Differences in population as well as the inhomogeneity of the definitions and diagnostic methods used probably explain part of the dispersion of observed prevalences.^[9]^ The diagnosis of SAS is based on a night recording, with either polysomnography or respiration polygraphy. However, in many countries, testing capacities are insufficient or non-existent and the resulting lack of diagnosis leads to insufficient management.^[9]^ The epidemiological studies available in the literature concerning this condition, come mainly from Western countries. In French-speaking sub-Saharan Africa, where resources are limited, there has been limited research on SAS diagnosis and management. This study was initiated to determine the prevalence of the main symptoms of SAS in the general population of Burkina Faso.

## Methods

### Framework and type of study

We conducted a descriptive and analytical cross-sectional study that took place at the Yalgado Ouédraogo University Hospital Center (CHU-YO), a reference centre in Burkina Faso. CHU-YO is located in Ouagadougou, the political and administrative capital of Burkina Faso. The population of Burkina Faso was estimated at 19 034 397 inhabitants in 2016, with 2 637 303 inhabitants in Ouagadougou.^[10]^

### Study population

The study targeted caregivers of outpatients in CHU-YO’s Departments of Medicine and Surgery. This study randomly included all men and women aged 25 and over, who gave their informed consent. People working night shifts were excluded.

### Data collection

The data were collected from 1 September 2014 to 31 October 2014. An anonymous survey sheet collected the sociodemographic characteristics, the main symptoms of the SAS, the sleep habits as well as the significant medical histories. The Epworth scale was used to evaluate daytime sleepiness and the Pichot scale was used for fatigue. The questionnaire was administered as a one-on-one interview and explained in the local language for patients who did not speak French. The measurement of anthropometric parameters was done by an intern (7th year medicine). Table 1 shows the operational definitions.

### Statistical analysis

Data were analysed using Epi Info 7.2 statistics software (CDC, USA). Pearson’s Gross χ² test or Fischer’s exact test was used to compare categorical variables when necessary. Mean values were presented with the standard deviation as the dispersion index. Associations between variables were considered statistically significant at the 0.05 probability threshold.

## Results

### Sociodemographic characteristics and medical history

The study involved 311 subjects whose characteristics are presented in Table 2. The population was predominantly male (58.2%) with a sex ratio of 1.43. The average (standard deviation (SD)) age of the subjects surveyed was 31.84 (8.25) years, with extremes of 25 and 70 years. More than half of them (59.2%) had a higher education level and 53.7% had a professional activity. The average (SD) body mass index (BMI) was 23.14 (3.67) kg/m², with extremes of 14.37 and 37.18 kg/m².

A history of hypertension was found in 3.8% and 14.5% consumed alcohol, while 6.4% were smokers. Of our respondents, 4.2% had had a non-sleep-related motor vehicle accident while driving. However, 5.1% were drowsy behind the wheel.

## Symptoms of SAS

Among our respondents, 17.7% felt that the duration of their sleep was not sufficient. On the Epworth scale, 30.5% of the subjects surveyed had a sleep deficit (EDSS score between 11 and 15) and 4.5% had excessive daytime sleepiness (EDSS score ≥16). The Pichot scale showed that 0.3% of our patients had excessive fatigue. Almost a quarter of our respondents (24.8%) usually snored. The existence of respiratory pause during sleep was found in 9.6% of our investigations. Nocturia was greater than twice per night in 11.9% of subjects. The prevalence of high suspicion of SAS was 9.6%. Subjects with high suspicion of SAS were older, had a higher mean BMI than the rest of the population, consumed alcohol and had a nocturia of more than two episodes a night. Factors associated with high suspicion of SAS are summarised in Table 3.

## Discussion

Sleep disorders are increasingly recognised as a real public health problem.^[1]^ Epidemiological studies of these conditions in the literature are mainly from Western countries.^[12]^ The management of SAS is recent in Burkina Faso, and it was necessary to initiate this study to determine the prevalence of the main symptoms of SAS in the general population of Ouagadougou. The average age of the surveyed subjects was 31.84 years, which is representative of the general young adult population of Burkina Faso. The present study had a prevalence of 9.6% of a strong suspicion of SAS. Studies in France and Morocco reported similar prevalence rates of 8.5% and 9.5%, respectively.^[9,12]^ In the literature, the prevalence of SAS in the adult population varies between 3% and 28%.^[1]^ The differences in population and the inhomogeneity of definitions and diagnostic methods used probably explain some of the dispersion of observed prevalences. The diagnosis of SAS is based on a night recording, with either polysomnography or ventilation polygraphy. These diagnostic confirmation tools are necessary in our context where resources are limited. The country has only two facilities (one in the CHU-YO pulmonology department and the other in a private clinic) to diagnose sleep-related respiratory disorders. In these structures, the diagnosis is made using respiratory polygraphy. The testing capacities are therefore insufficient with regard to the prevalence of symptoms of SAS. It is hence necessary to set up sleep laboratories to ensure adequate care.

In our study, strong suspicion of SAS was noted in 19 men and 11 women. SAS is 2 to 3 times more common in men than in women in the main cohorts, including the Wisconsin Sleep Cohort Study (WSCS), Southern Pennsylvania Cohort (SPC) and Victoria-Gasteiz Spain Cohort (VGSC).^[13–15]^ These studies indicate that the prevalence of SAS almost linearly increases among adults up to age 65, independently of other risk factors. As a result, these studies have confirmed the increase in the prevalence of SAS in overweight individuals.^[13–15]^ In our study, subjects with high suspicion of SAS were older, and had a higher mean BMI than the rest of the population.

We defined the association of ordinary snoring with daytime sleepiness and/or frequent apnoeas observed by a room sharer as ‘strong suspicion of sleep apnoea syndrome’. Indeed, snoring is an almost constant sign of SAS and it has been suggested that the diagnosis of SAS was most often made after a long history of snoring which became irregular and interspersed with apnoea.^[16]^ However, snoring does not necessarily mean SAOS.^[17]^ Snoring is very common in the general population; its prevalence increases in both sexes after age 35. It has been recognised for about four decades that 60% of men and 40% of middle-aged women (40 - 60 years) are habitual snorers.^[16]^ In our study, 24.8% of subjects usually snored. A prevarication bias commonly encountered in this type of study may explain the low proportion of snorers in our study.

The existence of excessive daytime sleepiness is also one of the major clinical criteria for diagnosing SAS.^[18]^ Here, we report 4.5% of subjects with excessive daytime sleepiness. Apnoeic subjects have nocturnal micro-awakenings that cause fragmentation and disorganisation of sleep. The quality of sleep is impaired because it becomes superficial and non-recuperative. This sleep debt is indirectly paid for by daytime sleepiness.^[17]^

Finally, the apnoeas documented in the literature are often a reason for consultation for unknown SAS carriers. The existence of respiratory pause during sleep was found in 9.6% of our respondents. These apnoeas are responsible for desaturations with sympathetic hyperactivity, excessive production of oxidants and exaggerated variations in intra-thoracic pressure at the origin of cardiovascular and metabolic disorders.^[19]^

However, our study did not aim to diagnose SAS by substituting for direct measurements of respiratory abnormalities during sleep. Indeed, none of the usual symptoms of SAS is specific enough to rule in the diagnosis.^[20]^ The combination of several symptoms, while improving the presumption, is also insufficient^[21]^ to establish the diagnosis of SAS. This study is certainly a first step on the estimated prevalence of clinical symptoms suggestive of SAS in the Burkinabe population.

## Conclusion

Our study showed that the prevalence of the usual symptoms of SAS is high in a country where the management of sleep-disordered breathing is still in its early days. The prevalence of high suspicion of SAS was 9.6%. Respiratory polygraphic and polysomnographic testing should be offered to presumptive cases for early diagnosis and treatment. A prevalence survey of SAS should be conducted nationally to assess the true extent of the problem in Burkina Faso.

## Figures and Tables

**Table 1:** Operational definitions

**Epworth Daytime Sleepiness Scale (EDSS)**^[11]^
- Score <11: normal vigilance
- Score between 11 and 15: Sleep deficit
- Score ≥16: Signs of excessive daytime sleepiness
**Pichot Fatigue Scale**
- Score ≤22: normal
- Score >22: excessive fatigue
**BMI**
- BMI <18.5: Underweight
- 18.5≤ BMI <25: Normal
- 25≤ BMI <30: Overweight
- BMI ≥30: Obese
**Strong suspicion of SAS **
- Existence of ordinary snoring (at least 4 nights per week)
- Daytime sleepiness
- and/or apnoea as observed by a person sharing the room (often or almost every night)

BMI = body mass index (kg/m²)

SAS = sleep apnoea syndrome

**Table 2:** Sociodemographic characteristics and medical history

**Variables**	**Frequency, *n *(%)**	
**Age group (years)**	
<30	170 (54.7)
30 - 39	99 (31.8)
40 - 49	24 (7.7)
50 - 59	13 (4.2)
≥60	5 (1.6)
**Gender**	
Male	181 (58.2)
Female	130 (41.8)
**Level of education**	
College	184 (59.2)
High school	73 (23.5)
Primary school	35 (11.2)
No formal school education	19 (6.1)
**Profession**	
Pupils/students	118 (37.9)
Functionary	100 (32.2)
Informal sector	45 (14.4)
Merchants	22 (7.1)
Housewives	21 (6.8)
Retired	5 (1.6)
**Body mass index**	
Underweight	12 (3.8)
Normal	231 (74.3)
Overweight	52 (16.7)
Obesity	16 (5.2)
**Past medical history**	
Alcohol	45 (14.5)
Tobacco	20 (6.4)
Hypertension	12 (3.8)
Depression	9 (2.9)
Asthma	8 (2.6)
Diabetes	1 (0.3)

**Table 3:** Factors associated with a high suspicion of SAS

		**High suspicion of SAS**				
**Variables**		**Yes, *n* (%)**	**No, *n* (%)**	**OR (95% CI)**	***p*-value**
Gender	Male	19 (10.4)	164 (89.6)	1.2 (0.6 - 2.7)	0.60
	Female	28 (63.6)	16 (36.4)		
Age (years)	≥45	11 (39.3)	17 (60.7)	9 (3.7 - 21.9)	0.00
	<45	27 (6.7)	264 (93.3)		
BMI (kg/m²)	≥25	12 (17.6)	56 (82.4)	2.7 (1.2 - 5.9)	0.012
	<25	18 (7.4)	225 (92.6)		
Sufficient sleep	No	12 (21.8)	43 (78.2)	3.7 (1.6 - 8.2)	0.00
	Yes	18 (7.0)	238 (93)		
History of hypertension	Yes	2 (18.2)	9 (81.8)	2.2 (0.3 - 9.6)	0.33
	No	28 (9.3)	272 (90.7)		
Tobacco	Yes	1 (5)	19 (95)	0.5 (0.1 - 2.8)	0.47
	No	29 (10)	262 (90)		
Alcohol	Yes	6 (30)	14 (70)	4.7 (1.5 - 13.3)	0.001
	No	24 (8.3)	267 (91.7)		
Daytime sleepiness	Yes	14 (100)	0 (0)	-	-
	No	16 (5.4)	281 (94.6)		
Snoring	Yes	18 (23.4)	59 (76.6)	5.6 (2.8 - 12.6)	0.000
	No	12 (5.1)	222 (94.9)		
Respiratory pauses during sleep	Yes	15 (50)	15 (50)	17.4 (7.2 - 43.1)	0.000
	No	15 (5.3)	266 (94.7)		
Pichot Scale	>22	1 (100)	0 (0)	-	-
	≤22	29 (9.4)	281(91.6)		
Nocturia	>22	9 (20.9)	24 (79.1)	3.1 (1.3 - 7.3)	0.007
	≤22	21 (7.8)	247 (92.2)		

SAS = sleep apnoea syndrome

OR = odds ratio

CI = confidence interval

BMI = body mass index
